# Supplementary figures and images for: Chronic loss of STAG2 leads to altered chromatin structure contributing to de-regulated transcription in AML
Source: J Transl Med. 2020 Sep 3;18:339. doi: 10.1186/s12967-020-02500-y (PMC7469420; doi:10.1186/s12967-020-02500-y)

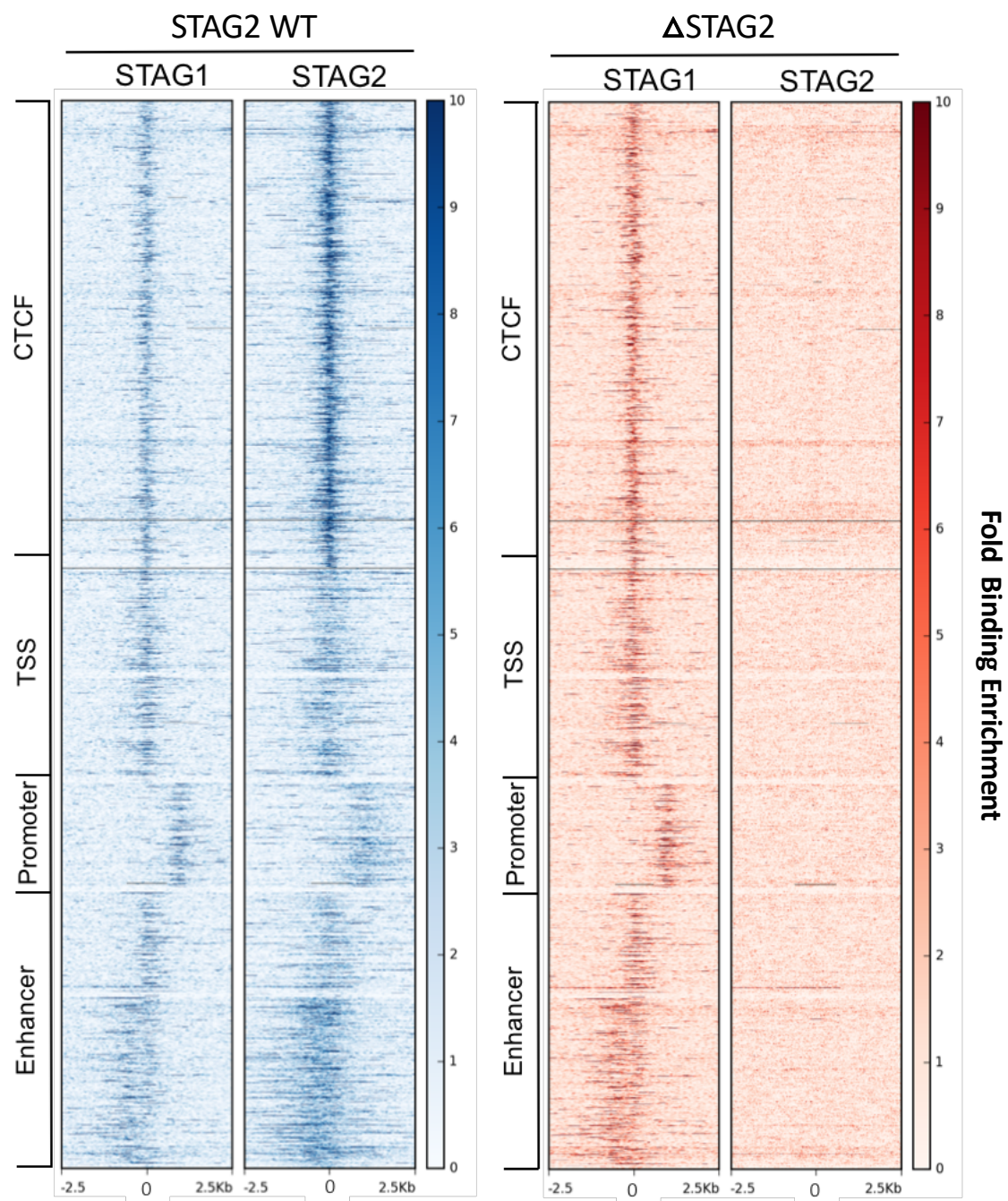

Figure S1

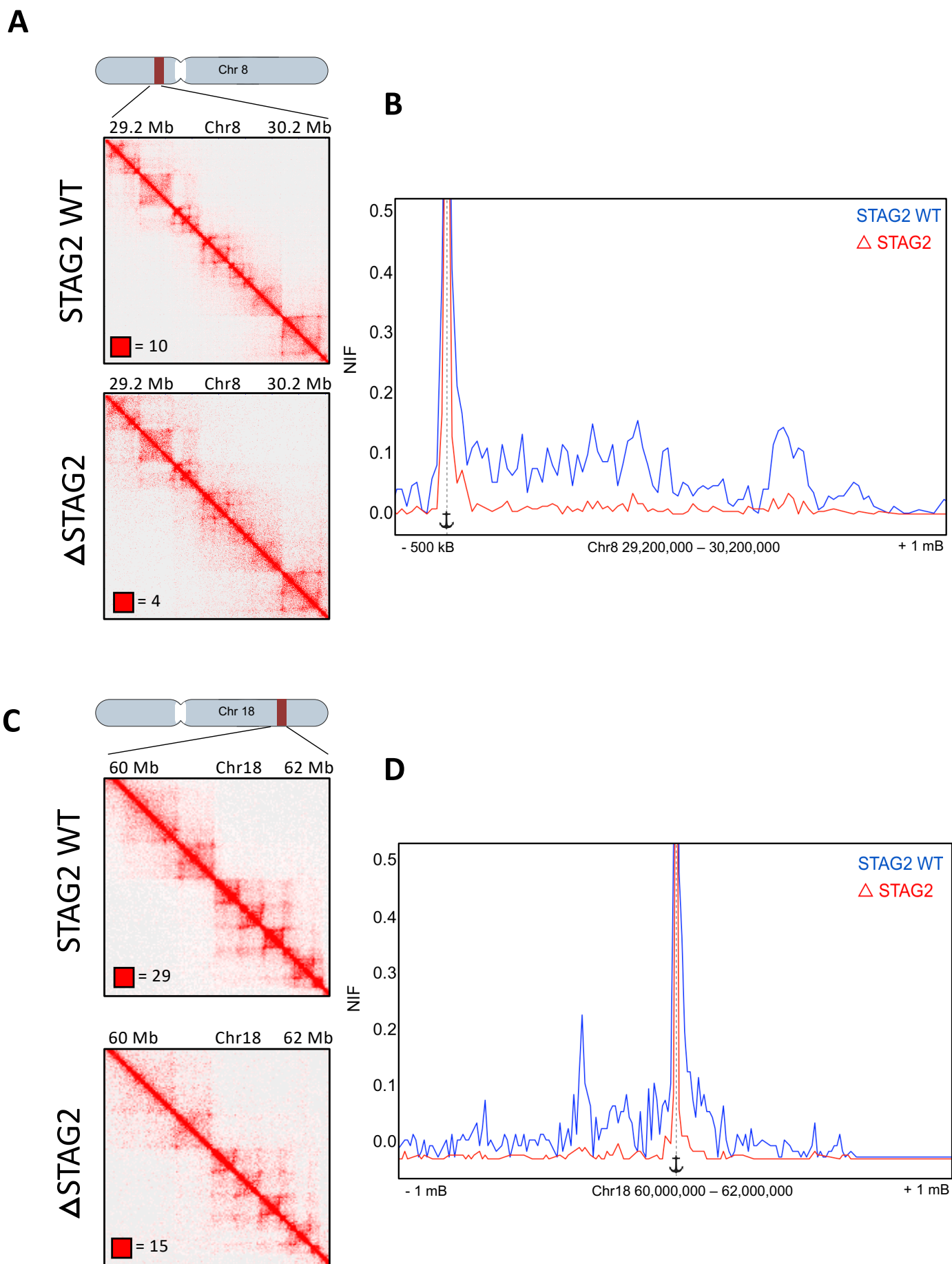

Figure S2

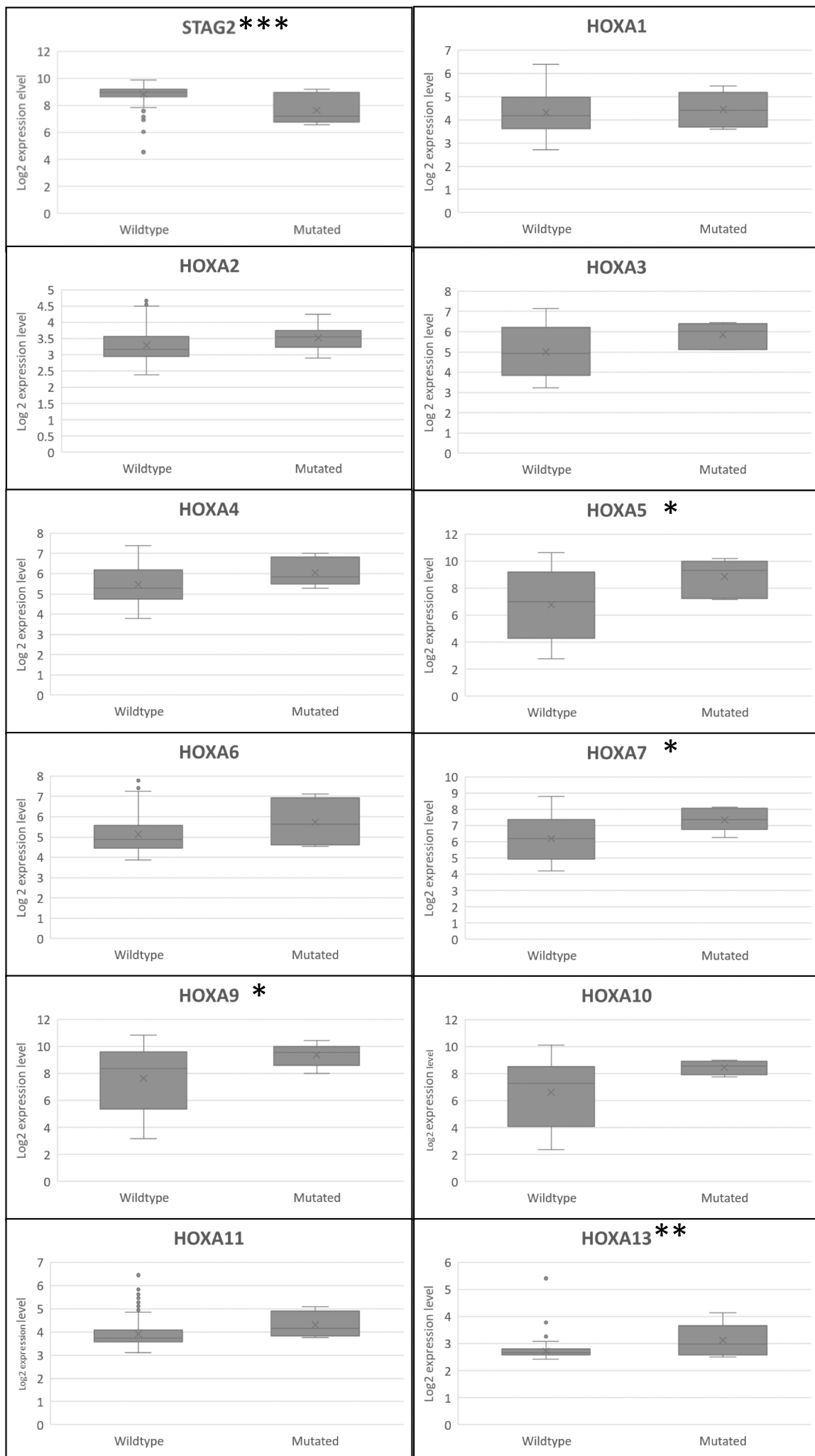

Figure S3

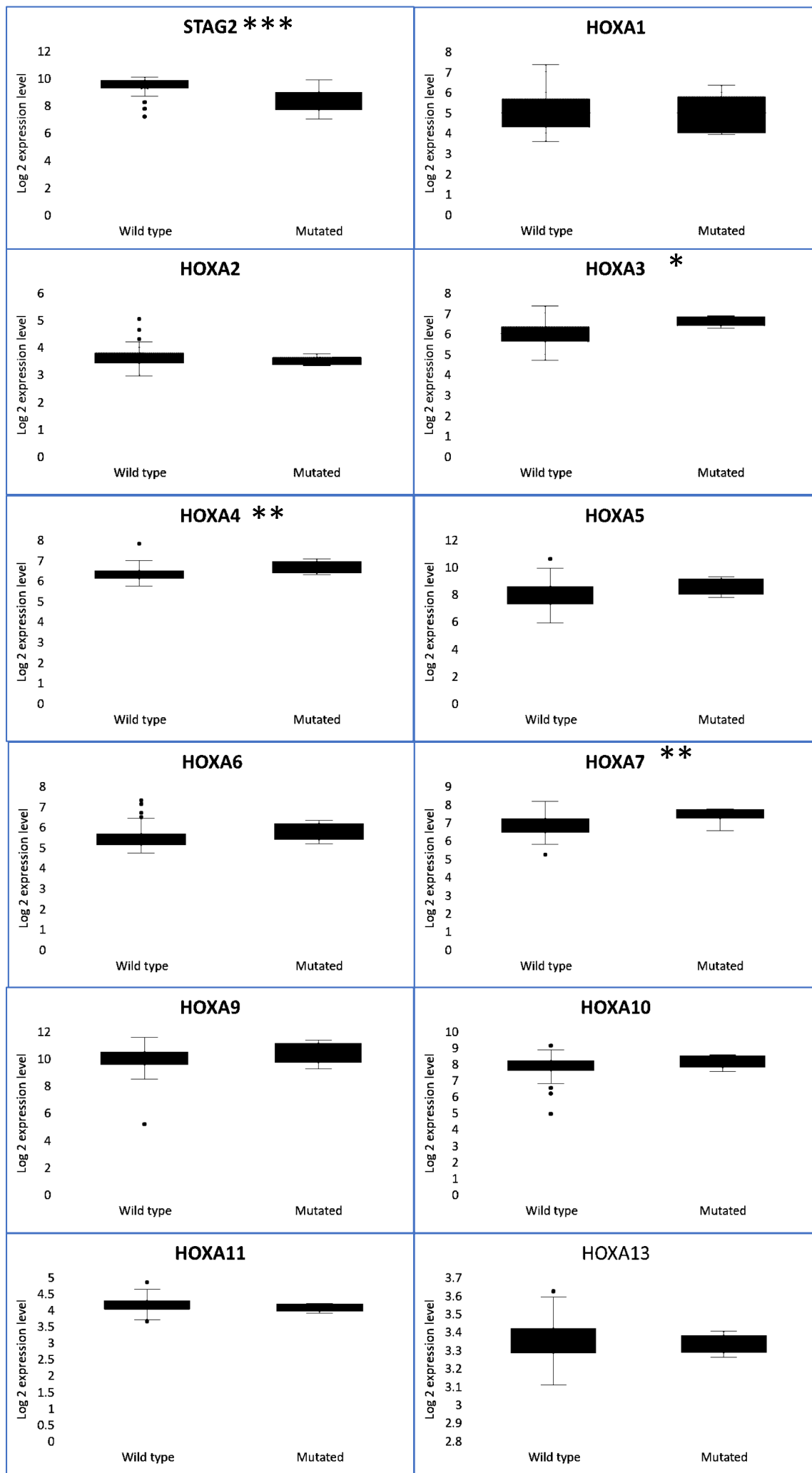

Figure S4

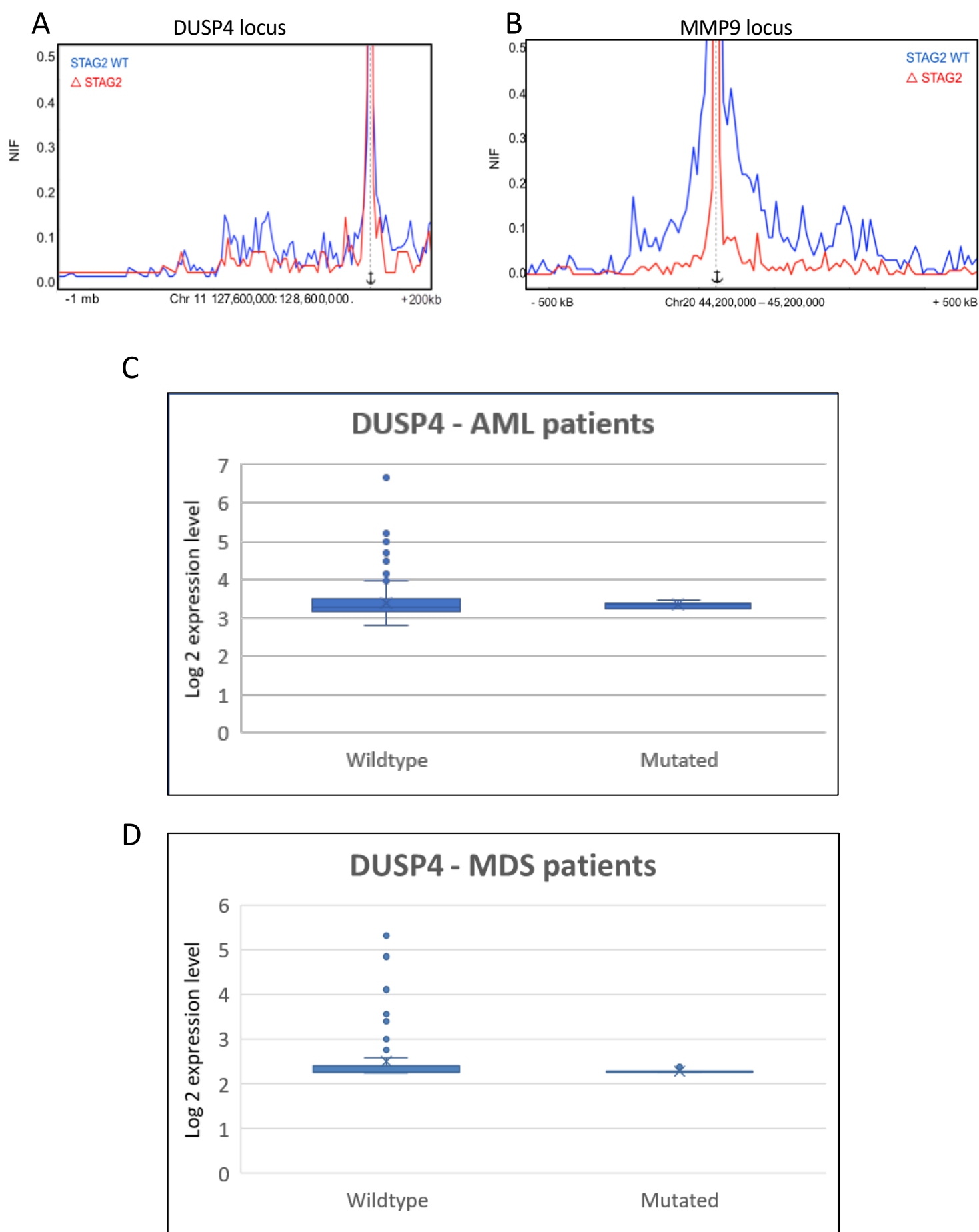

Figure S5

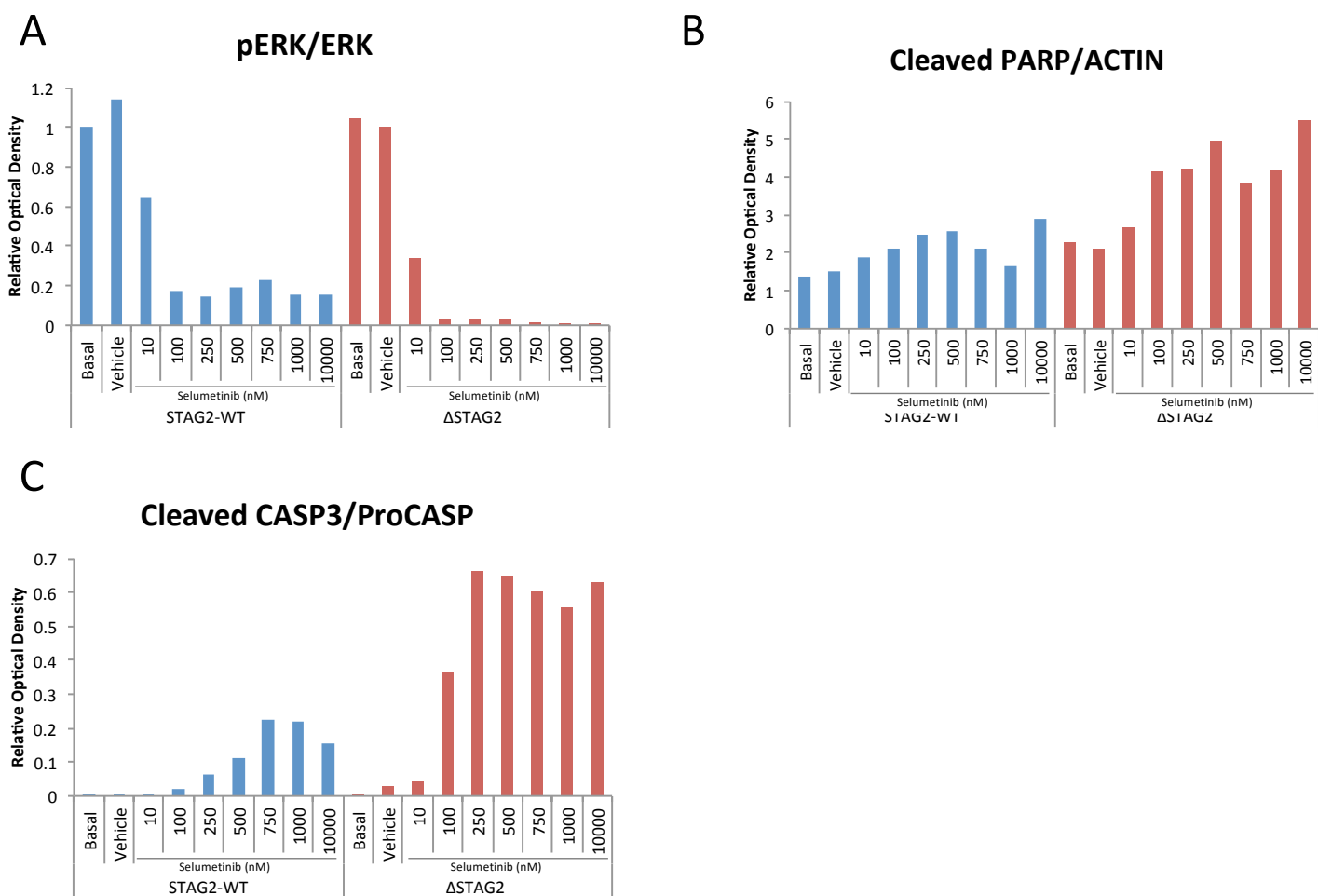

Figure S6

Supplement: Supplementary file 1 — Additional file 1: Figure S1. Genomic distribution of STAG1 and STAG2. Heatmap profiles of binding patterns for STAG1 and STAG2 across the genome in both STAG2-WT and ΔSTAG2 cells. Binding profiles are plotted from -2.5 – 2.5Kb either side of CTCF binding sites (CTCF), transcription start sites (TSS), promoters and enhancers. Colour scale depicts fold binding enrichment at these sites. Locations of all called binding peaks are provides in Table S1. Figure S2. Further examples of altered 3D structure within the ΔSTAG2 genome. a HiChIP Contact matrixes displaying interactions over a 1 Mb region 29.2 Mb into chromosome 8 (this region encompasses the DUSP4 gene). b Virtual 4C plot displaying interactions over the 1 Mb region depicted above. C HiChIP Contact matrixes displaying interactions over a 2 Mb region 60 Mb into chromosome 18 (this region encompasses the BCL2 gene). d Virtual 4C plot displaying interactions over the 2 Mb region depicted above. Figure S3. Altered gene expression in an AML patient cohort. Box and whisker plots of gene expression levels (log2) of STAG2 and genes in the HOXA locus between STAG2 mutant patients (n=6) relative to STAG2 wild-type (n=177) AML patients (GSE68833). Figure S4. Altered gene expression in an MDS patient cohort. Box and whisker plots of gene expression levels (log2) of STAG2 and genes in the HOXA locus between STAG2 mutant patients (n=6) relative to STAG2 wild-type (n=83) MDS patients (GSE58831). Figure S5. Altered chromatin structure surrounding MAPK signaling genes DUSP4 and MMP9 and DUSP4 expression in STAG2 wild-type and mutant patient samples. a Virtual 4C plot displaying interactions over a 1.2 Mb region of chromosome encompassing the DUSP4 gene. The V4c plot is anchored upstream of the DUSP4 gene. b Virtual 4C plot displaying interactions over a 1Mb region of chromosome encompassing the MMP9. c Box and whisker plots of gene expression levels (log2) of DUSP4 between STAG2 mutant patients (n=6) relative to STAG2 wild-type [file 12967_2020_2500_MOESM1_ESM.pdf]
